# Quantitative assessment of spasticity: a narrative review of novel approaches and technologies

**DOI:** 10.3389/fneur.2023.1121323

**Published:** 2023-07-05

**Authors:** Jian He, Anhua Luo, Jiajia Yu, Chengxi Qian, Dongwei Liu, Meijin Hou, Ye Ma

**Affiliations:** ^1^Research Academy of Grand Health, Faculty of Sports Sciences, Ningbo University, Ningbo, China; ^2^School of Information Management and Artificial Intelligence, Zhejiang University of Finance and Economics, Hangzhou, China; ^3^National Joint Engineering Research Centre of Rehabilitation Medicine Technology, Fujian University of Traditional Chinese Medicine, Fuzhou, China; ^4^Key Laboratory of Orthopaedics and Traumatology of Traditional Chinese Medicine and Rehabilitation (Fujian University of TCM), Ministry of Education, Fuzhou, China

**Keywords:** spasticity assessment, scales, portable devices, medical imaging, neuromusculoskeletal modeling, telemedicine

## Abstract

Spasticity is a complex neurological disorder, causing significant physical disabilities and affecting patients' independence and quality of daily lives. Current spasticity assessment methods are questioned for their non-standardized measurement protocols, limited reliabilities, and capabilities in distinguishing neuron or non-neuron factors in upper motor neuron lesion. A series of new approaches are developed for improving the effectiveness of current clinical used spasticity assessment methods with the developing technology in biosensors, robotics, medical imaging, biomechanics, telemedicine, and artificial intelligence. We investigated the reliabilities and effectiveness of current spasticity measures employed in clinical environments and the newly developed approaches, published from 2016 to date, which have the potential to be used in clinical environments. The new spasticity scales, taking advantage of quantified information such as torque, or echo intensity, the velocity-dependent feature and patients' self-reported information, grade spasticity semi-quantitatively, have competitive or better reliability than previous spasticity scales. Medical imaging technologies, including near-infrared spectroscopy, magnetic resonance imaging, ultrasound and thermography, can measure muscle hemodynamics and metabolism, muscle tissue properties, or temperature of tissue. Medical imaging-based methods are feasible to provide quantitative information in assessing and monitoring muscle spasticity. Portable devices, robotic based equipment or myotonometry, using information from angular, inertial, torque or surface EMG sensors, can quantify spasticity with the help of machine learning algorithms. However, spasticity measures using those devices are normally not physiological sound. Repetitive peripheral magnetic stimulation can assess patients with severe spasticity, which lost voluntary contractions. Neuromusculoskeletal modeling evaluates the neural and non-neural properties and may gain insights into the underlying pathology of spasticity muscles. Telemedicine technology enables outpatient spasticity assessment. The newly developed spasticity methods aim to standardize experimental protocols and outcome measures and enable quantified, accurate, and intelligent assessment. However, more work is needed to investigate and improve the effectiveness and accuracy of spasticity assessment.

## Introduction

Spasticity is one of many sensory-motor signs and symptoms that may be present following an upper motor neuron (UMN) lesion, causing significant clinical problems such as physical disabilities, abnormal gait or motor disorders (1, 2). Spasticity is accompanied by both positive symptoms (e.g., excessive muscle tonus, stretch reflex, clonus, and spasms) (3) and negative symptoms (e.g., incoordination, fatigue, weakness, and impaired motor control) (4), affecting patient's quality of daily lives and increase the financial burden on families.

In clinical practice, spasticity is defined as a velocity-dependent increase in tonic stretch reflexes with exaggerated tendon jerks resulting from hyper-excitability (5). According to Lance's definition, assessing spasticity depends on velocity-dependent stretch reflex using passive motions. However, other reflex mechanisms such as cutaneous or nociceptive could also contribute to increased muscle activations and are difficult to distinguish from the proprioceptive reflex mechanisms described by Lance (1). Another study defined spasticity as a sensorimotor control disorder due to damage to upper motor neurons that involves intermittent or persistent involuntary muscular activity (6). The definition is based on disordered sensorimotor control, which causing involuntary contraction or inappropriate activity of skeletal muscles, not rely on velocity-dependent or tonic stretch reflexes (6).

There is no consensus on valid and reliable clinical spasticity measures, due to the patient's neurophysiological complexity and peripheral changes (7). Measurements of spasticity include clinical scales (3), biomechanical assessment (8) and neurophysiological methods (9). Clinical scales are easy to use and are not restricted to additional tools (10). However, the clinical assessment of spasticity using scales solely depends on physical rehabilitation therapist experience. The reliability and validity of spasticity scale are questioned by researchers (3, 11–14).

Objective assessment of spasticity using biomechanical techniques such as isokinetic dynamometer or pendulum test is considered a valid measurement in multiple joints and makes it possible for standardization of assessment protocols (15–17). Biomechanical methods can measure joint motions and resistance changes at different angles and speeds during passive motions (18, 19). Muscle mechanics during active motions are not considered during spasticity assessment (4). Quantifying spastic muscles during voluntary contractions is important for investigating the disordered neuromuscular properties of the target muscle groups (20). A recent study assessed the voluntary activation properties of muscles by calculating peak torque, keep time of the peak torque, and rise time (16). However, there are few studies investigating spastic muscles under voluntary functional tasks. The relationship between the spasticity muscle tone and muscle voluntary activation remains unclear (16, 21).

The mechanisms of spasticity are investigated mostly from neurophysiological studies (9). Researchers use surface electromyography (sEMG) to analyze the responses of spastic muscle groups to active or passive movements or electrical stimulations (9). The shown by sEMG responses that muscle fiber conduction velocity, median frequency and mean power frequency are found smaller on the paretic side than on the unaffected side in patients with spasticity (4, 22). Other neurophysiological methods such as Hoffmann reflex (H-reflex) (9) and F-wave (23) were also used for spasticity measurement. H-reflex and F-wave involve excitability in the reflex arc and excitability in the alpha-motor neuron, respectively (23). The mean latency of H-reflex in patients ranked as 1 or 1+ using the Modified Ashworth Scale (MAS) is longer than in patients with MAS of 2 (24). The mean amplitude and mean duration of F-wave are significantly longer in patients with spasticity than in healthy patients (25). But H-reflex and F-wave are not used routinely in clinical practice due to the lack of standardized protocol and outcome indicator (23, 26).

Balci and Luo et al. reviewed previously used methods for spasticity assessment, such as clinical scales, gait analysis and neural and non-neural contribution measurements (4, 10). However, there have been many new developments that have not been summarized since 2016 due to the improvement of evaluation techniques and growing interest in spasticity research. New developments include new spasticity scales, medical imaging technologies, spasticity assessment devices, repetitive peripheral magnetic stimulation (rPMS), neuromusculoskeletal models and telemedicine-based spasticity assessments. The recently developed methods provide alternative or better solutions in spasticity assessment. Our review aims to investigate spasticity assessment approaches from 2016 to 2022, which can be used as spastic tools in clinics and provide valuable information for future spasticity research and assessment tool development. In addition, we believe this time period is appropriate because it allows us to capture recent advancements and innovations in spasticity assessment that have emerged over the past few years. However, we acknowledge the impact of the COVID-19 pandemic on research and development in various fields, including spasticity assessment. While it is true that our chosen time period covers the period affected by the pandemic, it is important to emphasize that the literature published during this time still offers valuable insights and contributions to spasticity assessment. In particular, this period has provided enough data and research to support our understanding of new spasticity assessment methods.

## Methods

We conducted a web-based search for relevant literature using the following electronic databases: Scopus, PubMed and Web of Science. We employed a query based on the following keywords (‘All fields' and MeSH): (1) spas^*^, (2) hyperton^*^, (3) measur^*^ or assess^*^ or evaluation (4) cerebral palsy or stroke or spinal cord injury or multiple sclerosis. The formal logical query was (1 OR 2) AND 3 AND 4. The search was conducted up until December 4th, 2022.

To be included, the screened papers had to satisfy the following criteria: (A) inclusion of the specified query in the abstract and/or title and/or in the keywords, (B) full-paper articles published in in peer-reviewed journals between 2016 and 2022, (C) availability in English, and (D) recruitment of human participants affected by spasticity conditions such as stroke, cerebral palsy, spinal cord injury and multiple sclerosis.

After removing duplicate articles, one author screened the remaining articles based on their titles and abstracts. Subsequently, two authors conducted a full-text screening to determine the eligibility of the remaining articles. In cases where the two authors disagreed on the results for the same article, a third author was involved in the evaluation process.

**Figure 1:**
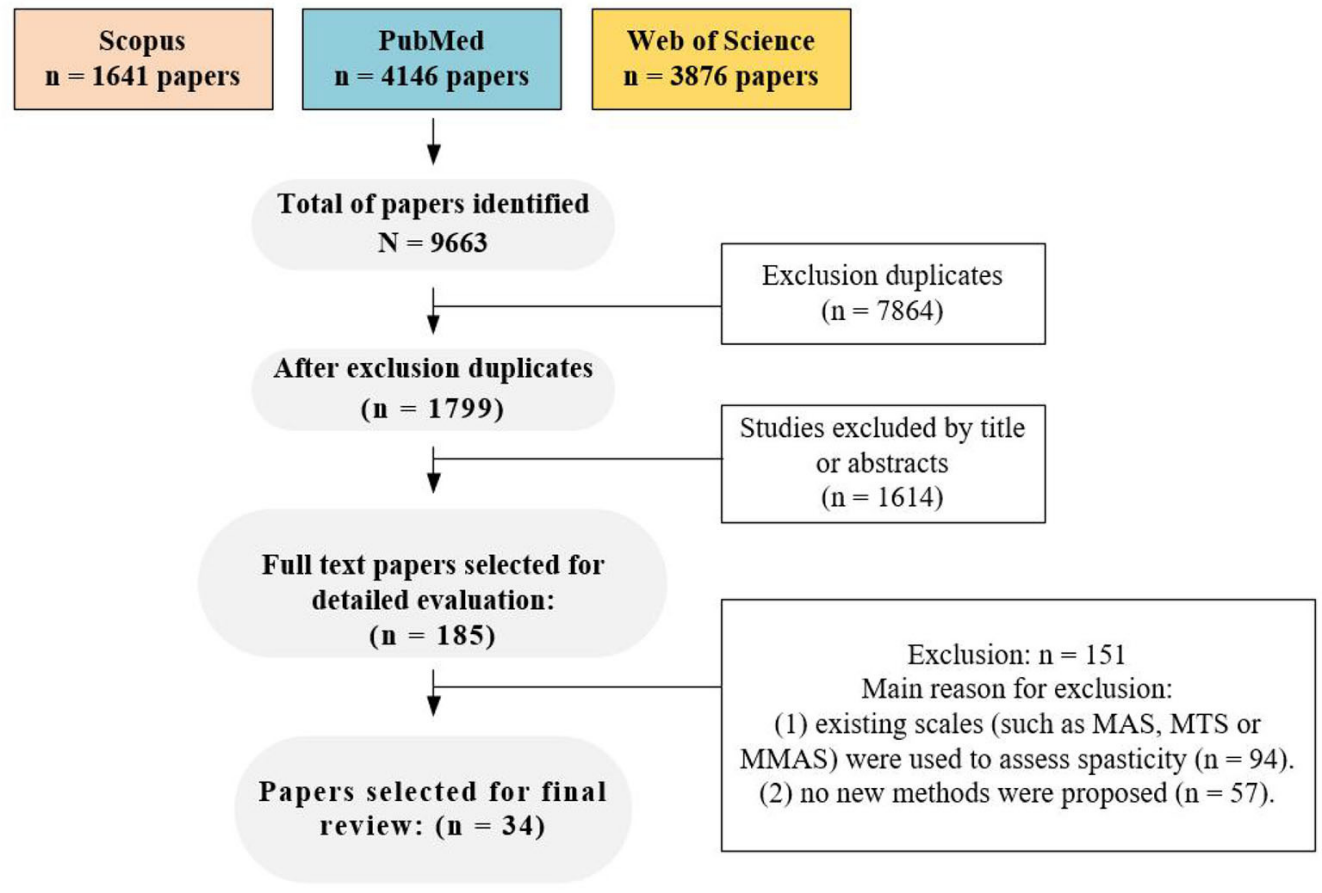
Flow chart of article search and selection strategy.

A flow chart depicting the selection process is presented in Figure 1. A total of 185 potential full-text articles were selected following keyword screening in the database, after accounting for duplicates articles and evaluating related titles and abstracts. Further screening of these full-text articles revealed that 94 articles used previous clinical scales such as MAS, MTS, or MMAS for spasticity assessment. Moreover, 57 articles presented concepts or trends without reporting new approaches, but only. Finally, 34 articles were included in the final analysis, and the spasticity methods proposed in the included studies were classified into six types (new clinical spasticity scale, medical imaging, spasticity evaluation device, magnetic stimulation, musculoskeletal model and telemedicine), which were discussed separately. Additionally, prior to the discussion, we conducted a review of the reliability studies of the commonly used clinical spasticity assessment methods.

## Reliability of current clinical spasticity assessment approaches

Most clinical spastic evaluations depend on changes in resistance during passive motions at constant such as Ashworth Scale (AS) (27), Modified Ashworth Scale (MAS) (28) or various stretch speeds such as Tardieu Scale (TS) (29), Modified Tardieu Scale (MTS) (30) and Composite spasticity scale (CSS) (31). Beside clinical scales, the pendulum test is also employed for spasticity assessment (15). The reliability of these clinical spasticity assessment methods in different studies has been shown to be moderate to good (see Table 1).

**Table 1:** Reliability of commonly used clinical scales in spasticity assessment.

**Scales**	**Muscle groups**	**Reliability**	**References**
AS	HAM; GAS	Three raters assess (ICC = 0.54~0.78)	(32)
	EF; KE	Three raters assess EF (ICC = 0.58) and KE (ICC = 0.63)	(33)
MAS	HAM; GAS	Three raters assess (ICC = 0.61~0.87)	(32)
	HA; KE; KF; APF	Two raters assess (ICC = 0.41~0.73)	(34)
MMAS	KE	Two raters use Cohen kappa test (*k =* 0.72~0.82)	(35)
	WF	Two raters use quadratic weighted kappa test (*k =* 0.92)	(36)
TS	EF; APF	Related to laboratory measurement EF (*r =* 0.86) and APF (*r =* 0.62)	(29)
MTS	EF; APF	Rater assess it twice (ICC>0.85)	(37)
	ADF; KE; HA	Two raters assess (ICC = 0.7)	(38)
CSS	SOL; APF; AD	With SR and EMG co-contraction ratios highly consistent (r>0.89)	(31)
	EF	With SR threshold of EF spastic negative correlation (*r =* −0.65)	(39)

AS, Ashworth Scale; MAS, modified Ashworth Scale; MMAS, modified modified Ashworth Scale; TS, Tardieu Scale; MTS, modified Tardieu Scale; CSS, Composite spasticity scale; HAM, hamstrings; GAS, gastrocnemius; EF, elbow flexor; KE, knee extensor; HA, hip adductors; KF, knee flexor; APF, ankle plantar flexors; WF, wrist flexor; ADF, ankle dorsiflexor; SOL, soleus; SR, stretch reflex; EMG, electromyography.

An examiner moves patient's joint quickly to rate the level of resistance and then spasticity level for the target muscle group using AS and MAS (18). The AS and MAS are rated on a 0–4 scale, with the MAS having an extra score of 1+, which is described as slight increase in muscle resistance throughout the range of motion (40). However, AS demonstrates significant variability among raters (33). In addition, MAS in the scoring system and related terminology is vague (28). An updated version of MAS, called Modified Modified Ashworth Scale (MMAS), is published (11). MMAS removed Grade 1+ and redefined Grade 2. By comparing the reliability of AS and MAS, it is found that the reliability of AS in measuring spasticity is poor (11). The Kappa values for AS is 0.17 (SE = 0.21, *p* = 0.41) in elbow flexors (11). It is necessary to discontinuing the use of AS to assess spasticity (33). MAS shows moderate to good reliability in the hip flexors (ICC = 0.61~0.87) (32). The reliability of MMAS in assessing knee extensor spasticity of patients with post-stroke is more reliable in comparison with AS and MAS, with Kappa values of 0.72~0.82 (41).

TS and MTS take into account passive range of motion (PROM) and muscle responses to passive stretch at the possible fastest stretching rate (30). TS and MTS measure spasticity using two parameters: the spasticity angle at different stretching speeds (V1, V2, and V3) and the spasticity grade. The angle is the difference between the angles of arrest at slower than the natural drop of the extremity segment due to gravity effect (V1) and of catch-and-release or clonus at as fast as possible (V3), but V2, which is the velocity of the limb segment naturally falling under gravity, is only practical in severely paretic patients (10, 42). Spasticity grade [0–5] is an ordinal variable that grades the intensity and thus measures the gain of the muscle reaction to fast stretch (42). A grade of 0 represents no resistance during passive motion; 1 represents minimal resistance during passive motion; 2 represents a clear catch at a precise angle, followed by release; 3 represents fatigable clonus occurring at a precise angle lasting < 10 s under pressure, followed by release; 4 represents unfatigable clonus occurring at a precise angle lasting >10 s under pressure occurring at a precise angle; and 5 represents the joint cannot be moved. MTS as an updated version of TS that increases extremities evaluation positions and the relative difference between slow and fast passive stretching determines the dynamic component of muscle contracture (43–45). A study evaluated the intra-rater reliability of MTS in assessing elbow flexors and ankle plantar flexors of adult stroke patients via angle of muscle reaction (R1), passive ROM (R2) and dynamic component (R2-R1) (37). The results showed MTS has very good reliability in R1, R2, and R2-R1 (ICC = 0.847). MTS is more appropriate than AS or MAS due to the velocity-dependence in assessment protocol (29, 45). However, TS and MTS takes slightly longer time during evaluation spasticity than that AS and MAS (29). MTS is also difficult to identify clonus reliance at the higher end of tones, and may exacerbate clonus after an intervention (46).

The CSS has been shown better to describe plantarflexor spasticity and to correlate with stretch reflex areas in adults with hemiplegia (47). CSS assess the three clinical indicators, which involves scores of tendon jerk, resistance and clonus, respectively (31). Adding these three scores provided composite spasticity scores ranging from 0 to 9, 10 to 12, and 13 to 16. This corresponds to mild, moderate and severe spasticity, respectively. When evaluation typically developing children and children with spastic CP, CSS was found to be highly consistent (*r* > 0.89) with stretch reflex, M-response areas, and EMG co-contraction ratios during ankle dorsiflexion (31). Additionally, a significant negative correlation (*r* = −0.65, *p* < 0.05) was found between elbow flexors spasticity and stretch reflex threshold using CSS measurements (39).

The pendulum test, which is often applied to the knee extensor muscles (e.g., the quadriceps), evaluates spasticity by observing a muscle's response to a rapid stretch imposed by gravity and the resulting oscillations between flexion and extension (18). The pendulum test is subjective, simple, quick to implement, reproducible, non-invasive, and non-intimidating to patients with cognitive impairments (48). A recent study used pendulum test to investigate the relationship between quadriceps spasticity and gait abnormalities in children with cerebral palsy (CP) (15). The results showed that swing excursion and relaxation index based on the pendulum test could differentiate the level of knee extensor spasticity in children with CP. The test-retest and inter-rater reliability of pendulum test are good in the children with spasticity CP and in the typically developing children (ICC = 0.79~0.95 and 0.88~0.99, respectively) (15).

## Recently developed clinical spastic scales

Scales are preferred methods in clinical spasticity assessment, even though the reliability of scales has been questioned by researchers (49). A series of new scales (50–53), including more information related to spasticity, are developed to improve the effectiveness of clinical spasticity assessment (see Table 2).

**Table 2:** Clinical spasticity scales developed in recent years.

**Scales**	**Compared tests**	**Subjects (sample size)**	**Muscle groups**	**Results**	**Characteristics**	**References**
NRS	AS; MAS	ND (23)	/	Validity of NRS is supported by a consistent association with PGIC scores; mean scores of NRS are high than MAS (4.03 vs. 1.30)	Combined with Patients use self-reports	(50)
SPAS	MAS	SCI (6)	QUA; HAM	SPAS and MAS are correlated; SPAS has more precise gradation	Based on quantitative data from two inertial and two EMG units during pendulum test	(52)
TSS	MAS; MTS	Stroke (84)	EF; WF; APF	Agrees with MAS and MTS (*r =* 0.840~0.946, *r =* 0.715~0.795); TSS scores are higher during standing	Muscle tone change at different stretch rates, clonus; dynamic muscle length	(51)
ASAS	MAS; MTS; TS	ABI (16) CP (22) Stroke (85)	SOL; BIC; WE; WF; TB-P; HAM; GAS; APF	ASAS have a good reliability between muscle groups (*k =* 0.75 ~0.92) and between raters (*k =* 0.87)	Combined merits of TS and MTS; ASAS confirming a velocity-dependent increased response to rapid passive movement	(53–55)

NRS, numeric rating scale; SPAS, SPAsticity scale; TSS, triple spasticity scale; ASAS, Australian spasticity assessment scale; AS, Ashworth Scale; MAS, modified Ashworth Scale; MTS, modified Tardieu Scale; TS, Tardieu Scale; ND, neurological disorders; SCI, spinal cord injury; CP, cerebral palsy; ABI, acquired brain injury;/, no description; QUA, quadriceps; HAM, hamstrings; EF, elbow flexor; WF, wrist flexor; APF, ankle plantar flexor; GAS, gastrocnemius; SOL, soleus; BIC, biceps; WE, wrist extensor; TB-P, tibialis posterior; PGIG, Patient Global Impression of Change; EMG, electromyography.

Patients may use different terms than clinicians to describe spasticity in clinical practice (56). Clinicians need to gather information from those patients to differentiate neurological sensations from specific spasticity symptoms (18). The Numeric Rating Scale (NRS) is developed to collect information about spasticity from the patient's perspective and is a great self-reported clinical assessment tool. The NRS rates spasticity from 0 to 10 based on patients' subjective perception, in which 0 stands for no spasticity and 10 stands for the severe spasticity (50). A recent study used NRS to assess patient's self-reported pain and spasticity and found that the prevalence of spastic disorders can be reported using NRS (2). Additionally, the test-retest reliability of NRS is considerably better than that of AS (ICC 0.83 vs. 0.53) for assessing multiple sclerosis (MS) patients with spasticity (57).

The SPAsticity Scale (SPAS) is developed based on torque measurements during pendulum test (52). The data used to calculate SPAS were collected by two inertial sensors and two sEMG recording units. Based inertial information and sEMG signals, SPAS defines two parameters a and b involving spastic torque resulting from involuntary reflexive activation of paralyzed muscles during the pendulum test (52). Parameter a correlates with the strength of the torque and b with the duration of the relaxation to the neutral position (52). The results suggested that SPAS and MAS are correlated in six subjects. Interestingly, SPAS give a more precise gradation (SPAS involving rational number, MAS involving integer) for spasticity assessment due to objective measure. However, the reliability of SPAS is unknown, studies involving most sample sizes and randomized control trial (RCT) are necessary to evaluate the effectiveness of SPAS.

The Triple Spasticity Scale (TSS) takes into account movement speeds, clonus states and dynamic muscle length to capture mild change in spastic limbs (58). TSS includes the following three subsections: (1) the increased resistance, which is scored according to two stretches (very slow (r2; < 5°/s) and as fast as possible (R1)) and the increased speed (R1–R2), which is scored according to the assessor's perception; (2) clonus, which is divided into three levels according to the sustained time of the clonus; and (3) dynamic muscle length, also known as angle difference between R1 and R2, which is converted into 5 grades (58). TSS scores range from 0 to 10, in which 0 indicating no spasticity and 10 representing severe spasticity. The test-retest reliability of TSS total score is good (ICC = 0.905~0.918) (51). Moreover, MTS and TSS scores are similar (*r* = 0.840~0.946, *p* = 0.000) in upper limb spasticity when evaluating different body positions such as sitting and standing in patients with post-stroke hemiplegic (51). TSS is also more sensitive to standing posture changes in spastic hemiplegia such as TSS scores are significantly higher in standing than sitting (*P* < 0.05) (51).

Australian Spasticity Assessment Scale (ASAS) takes into account the velocity –dependent features of TS and MTS and uses the similar scoring framework to MAS for clinical compliance (54). ASAS only considers two factors, the presence or position of the “catch” and the presence of resistance after the capture (55). ASAS has a prescribed test procedure, is quick and easy to perform, and has an unambiguous five-grades scoring system (54). ASAS has found good reliability (ICCs = 0.86~0.90) in its assessment of upper and lower limb spasms (53). Additionally, the inter-rater reliability of ASAS in adult stroke patients with spasticity is good in elbow flexors (kappa = 0.77~0.87), wrist flexors (kappa = 0.72~0.82) and ankle plantar flexors (kappa = 0.72~0.85) (55).

Clinical scales are the preferred method of spasticity assessment in a simple and convenient manner, especially MAS and MTS are used more frequently in the clinical evaluation of spasticity than spasticity scales. It is now widely used in different settings such as outpatient and scientific research. Although these clinical scales have reported good reliability in the assessment of spasticity, a combination of objective methods such as instrumentation is necessary.

## Medical imaging based spasticity assessment

Medical imaging technologies, such as near-infrared spectroscopy (NIRS) (59), magnetic resonance imaging (MRI) (60), quantitative ultrasound technology (QUS) (61) thermography (62) and spasticity scale based on ultrasonography (63, 64), are used to assess spasticity (see Table 3).

**Table 3:** Medical imaging methods based on spasticity assessment.

**Methods**	**Compared tests**	**Subjects (Sample size)**	**Muscle groups**	**Results**	**Characteristics**	**References**
NIRS	MAS	Stroke (23)	FAF	Spastic muscles the pattern of change was not the same for (tHb)and (O_2_Hb) compared normal muscle same direction	Detect blood volume and oxidative capacity changes	(65)
MRI	MAS	CP [TB (45)] [PB (48)]	Lower limb muscles	Differentiate preterm spastic types and neuroimaging patterns using MRI scanning	Compare the cross-sectional area and volume of spasticity muscles	(60)
PI	MAS	Stroke (24) Normal (16)	BBM	Spastic muscle appears more echogenic and higher PI than healthy muscle	Measure morphology, structure and echogenicity of muscles	(61, 66)
USI	MAS; TS	Stroke/normal (7/8) Normal (10)	BBM	Strain rates of spasticity muscles is lower; intraobserver reliability is good in stroke patients (*r =* 0.85); good reliability in BBM (ICCs>0.75)	The strain of USI varies with the deformation of spastic tissue	(67, 68)
SWE	MAS; MTS	Normal (19) Stroke/normal (31/21)	BBM; QM	Faster through stiff tissues; SWV increases with the level of spasticity; good reliability in BBM and QM (ICC > 0.75)	Measure mechanical properties of tissues	(69, 70)
TG	MAS	Stroke (40) Stroke (100) Pemiplegia (33)	Lower limb muscles	Temperature of spasticity side is lower; temperature is affected by level of spasticity	Observe changes of temperature in spasticity muscles	(62, 71, 72)
HS	CMFCS	CP (60)	GAS; SOL	Weak correlation to GMFCS; moderate inter-rater reliability (kappa = 0.60~0.73).	Evaluate echo intensity of muscles	(63)
MHS	MAS; UI; GS	Spasticity (45) Normal (5)	Muscles affected by spastic	Significantly correlates with GS (*r =* 0.829); excellent reliability (ICCs = 0.76~0.81)	Grades 2 and 3 are clearly defined	(64)

NIRS, near-infrared spectroscopy; MRI, magnetic resonance imaging; PI, pixel intensity; USI, ultrasound strain imaging; SWE, shear wave elastography; TG, Termography; HS, heckmatt scale; MHS, modified heckmatt scale; MAS, modified Ashworth Scale; TS, Tardieu Scale; MTS, modified Tardieu Scale; CMFCS, gross motor function classification system; UI, ultrasound imaging; GS, grayscale scores;CP, cerebral palsy; TB, term born; PB, preterm born; FAF, forearm flexors; BBM, biceps brachii muscle; QM, quadriceps muscle; GAS, gastrocnemius; SOL, soleus; tHB, total hemoglobin; O_2_HB, oxygenated hemoglobin; SWV, shear wave velocity.

NIRS is a non-invasive optical technique for detecting real-time muscle hemodynamics and metabolism (73). The widely used outcome measures of NIRS include oxygenated hemoglobin (O_2_Hb) concentration, deoxygenated hemoglobin (HHb) concentration, total hemoglobin (tHb) concentration, and the tissue oxygenation index (TOI%), (an index of local tissue oxygenation calculated from O_2_Hb and HHb) (59). In addition, NIRS can detect blood volume differences and oxidative capacity changes between normal and dysfunctional muscles (74). NIRS is easy to use in clinics (59). Although lack of high-quality RCT studies, preliminary results have shown that NIRS correlates with other spastic measures such as the MAS and sEMG (59). However, it is important to note that NIRS can provide direct information on muscle metabolism, but its measurement depth is limited to the superficial layer of muscle tissue (75, 76). Therefore, due to the limited of the measurement depth of the NIRS technique, its relationship with the MAS scale and the myographic electromyographic signal is only applicable to superficial muscles. For deep muscle research, other techniques or methods are needed.

MRI can provide information on muscles' elastic parameters and changes in muscle tissue properties before and after an exercise (77). A standard MRI scanner applies mechanical vibration to muscle via the skin, creating shear waves that penetrate the tissue and propagate along muscle fibers (77). Spasticity can be assessed by comparing the cross-sectional area and volume of spasticity muscle using MRI (61). For example, when comparing children with CP and adult with spasticity hemiplegia, a reduction in the volume of lower extremity muscles that are more affected by spasticity was found (78). Additionally, MRI can be used to identify various neuroradiologic patterns in children with spastic diplegia (60). A higher-resolution MRI could reveal previously unnoticed abnormalities in these children, particularly when using more sophisticated imaging protocols (60).

QUS techniques, including pixel intensity (PI) of grayscale image (61), ultrasound strain imaging (USI) (67), and shear wave elastography (SWE) (79), can identify the echogenicity and mechanical properties of normal and spastic tissues. PI of grayscale image can evaluate muscles' morphology, structure, and echogenicity by quantifying tissue echogenicity using computer-aided computation (61). Spastic muscles appear more echogenic and have a higher PI in comparison with healthy muscles (80). Additionally, the PI of grayscale image values is also significantly higher in the post-stroke spastic biceps brachii muscle than those in post-stroke non-spastic and healthy biceps brachii muscles (66). This result is consistent with results of Stecco et al. (80). However, the correlation between PI of grayscale image values and MAS and TS scores for biceps brachii muscle are poor (R2 = 0.01, *p* = 0.95) (66).

Quantitative echogenicity alone cannot assess the mechanical properties of the spastic muscles, muscle's architectural parameters, such as muscle thickness, fascicle length and pennation angle, have a closer relation to spasticity (61, 81). USI may be a better choice. USI is defined as the strain caused by ultrasound transducers or other devices compressive force on tissue (82). However, a variety of strain with the depth of tissue deformation (61). Strain is higher in softer tissues because it can withstand greater deformation and is lower in stiff tissue due to limited deformation. A recent study used USI to compare the strain of spasticity muscles (e.g., biceps brachii) to those of healthy muscles (67). USI has a good inter and intra-observer reliability in the assessment of biceps brachii muscle spasticity (ICCs > 0.75) (68). It is worth noting that USI focus on spasticity muscles on the upper limbs and lack of studies on lower limb muscle groups.

SWE also can be used as a tool for measuring the mechanical properties of tissues (61). Measurements of SWE often use propagated waves (also called shear waves), produced by ultrasound push pulses when operation (83), traveling laterally and perpendicular to the transducer's acoustic ultrasound waves (84). Shear waves travel more easily longitudinally along muscle fibers than to perpendicularly (85) and also faster through stiff tissue than soft tissue (86). The identification of spasticity by SWE is firstly established by quantifying muscle stiffness (61) such as a greater shear wave velocity (SWV) in stiff biceps (69). A recent study found that SWV is increased when the ROM of a spasticity muscle decreased (66). Additionally, a positive correlation was found between SWV and MTS and MAS (R2 = 0.662, *P* < 0.001 and R2 = 0.536, *P* = 0.002), and also found that SWV increases with the increased level of spasticity (70).

Thermography measures infrared radiation emitted by the surface of the body being studied (87). Lower limbs of patients with hemiplegia have lower temperatures than healthy subjects (71). The temperature of normal tissues, including skin, is affected by spasticity in pathological tissues. Therefore, the temperature of the spasticity limb is lower than the non-spasticity side (62). A new dynamic thermography technique assesses the quadriceps during a static load for healthy subjects and patients with spastic quadriplegia (72). The results found that the local temperature at the end of muscle contraction an increased in healthy subjects, and decreased in patients with spasticity (72). However, thermography is limited to the specific testing environment (such as controlled indoor temperature and air flow speed) (62).

The scale based on medical imaging has brought new findings for the assessment of spasticity. The Heckmatt Scale (HS) visually evaluates spasticity by echo intensity (EI) of the spastic muscles in the transverse view using a B-type real-time ultrasonography with a linear probe (63, 88). The spasticity is graded semi-quantitatively according to HS, Grade I is normal, grade II represents an increase in EI while bone echo is still distinct, grade III indicates a marked increase in muscle EI with a reduced bone echo and grade IV indicates a very high muscle EI and a complete loss of bone echo (89). This result prove that the echogenicity of the muscle measured with the HS is related to the diagnostic nerve block (DNB) outcomes confirming the relationship between the echogenicity and rheological muscle properties and between DNB and spasticity. To a more Heckmatt grade relates a lower DNB outcome. This is in accordance with the ability in differentiating contracture from the spasticity of the two methods. HS has moderate inter-rater reliability (kappa = 0.60~0.73) in assessment spasticity muscles (63). However, HS is difficult to differentiate spasticity at Grades 2 and 3 since muscles normally are not homogenously affected throughout its length, hindering precise distinctions (90). Modified Heckmatt Scale (MHS) is developed to obtain greater precision between grades, especially for muscles with mild to moderately increased EI (e.g., Grades 2 and 3) (64). MHS had moderate inter- and intra-rater reliability in assessing muscle echogenicity for patients with upper and lower limb spasticity (ICCs = 0.76~0.81) and was consistent with quantitative grayscale scores (*r* = 0.829, *p* < 0.001) (64). However, it should be noted that although the MHS is widely used for spasticity, its validation was conducted only 2 years ago. The validation of MHS holds significant value for advancing spasticity assessment and treatment. Additionally, information about nerve blocks is also provided, which can contribute to a better understanding of spasticity pathology.

Medical imaging techniques enable physicians to rely less on subjective tests such as MAS and MTS when managing spasticity. Medical imaging is feasible to provide quantitative information in the assessment and monitor the treatment effects of muscle spasticity (61). It is noteworthy that although medical imaging techniques can assess the properties of spasticity muscles, they do not seem to be able to quantify their resistance changes. Therefore, it is necessary to combine with other methods to quantify tension when using medical imaging techniques to evaluate spasticity.

## Spasticity assessment devices

Developing tools suitable for spasticity evaluation is a goal that clinicians or researchers are committed to. Many spasticity evaluation devices are developed such as portable-sensor based devices (49), robots-assisted equipment (91) and myotonometry (92) (see Table 4).

**Table 4:** Devices used for spasticity assessment in recent years.

**Devices**	**Compared tests**	**Subjects (sample size)**	**Muscle groups**	**Results**	**Characteristics**	**References**
Portable-Sensors	EMG; TSRT	Stroke/normal (16/10)	EF; EE BBM	Strong correlations with clinical scales (*r =* 0.86) and TSRT (*r =* −0.89)	Record data of spasticity limbs when stretched at low, moderate and high velocities	(93)
	MAS; ST	CVA/SCI (45/3)	EF; EE;	Up to 95.4% for accuracy	Machine-learning algorithms; monitoring spasticity changes	(94)
	MTS	CP (28)	APF; KF KE	Good test-retest and inter-rater reliabilities (ICC>0.8).	Monitoring spasticity changes; small size; high accuracy	(95)
Robot-Aided	MTS; ROM	Normal (15)	WF; WE	Promising in assessing wrist spasticity	Allows three degree of freedom wrist movements	(91)
	MAS; EMG	Stroke (5)	EF; EE	Detect changes in MAS; recording spasticity parameters	Automatically assessing spastic muscles during active and passive motions	(96)
	MAS; MTS; GMFCS	CP (16)	HF; HE KF; KE	Inter-tester ICC 0.32~0.70; better reliability at fast and medium speeds	Moves the lower limb at a controlled velocity; recording joint resistance	(97)
Myotonometry	MAS; ST	Hemiplegia (14)	BBM	Negative correlation with ST (*r* < −0.5)	High sensitivity	(92)
	ROM; ST	CP/MS/normal (9/8/8)	AF	Inter- and intra-rater reliability in all groups (ICC = 0.62~0.91)	Recognize spasticity by short pulses not depend on resistance changes in stretching	(98)

EMG, electromyography; TSRT, tonic stretch reflex threshold; ST, Stretch test; MAS, modified Ashworth Scale; MST, modified Tardieu Scale; ROM, range of motion; GMFCS, gross motor function classification system; CVA, cerebral vascular accident; SCI, spinal cord injury; CP, cerebral palsy; MS, multiple sclerosis; EF, elbow flexor; EE, elbow extensor; BBM, biceps brachii muscle; APF, ankle plantar flexor; KF, knee flexor; KE, knee extensor; WF, wrist flexor; WE, wrist extensor; HF, hip flexor; HE, hip extensor; AF, ankle dorsiflexor.

Portable-sensor based spasticity assessment devices are generally developed based on angular (99), inertial (95), torque (93) or sEMG sensors (49). Inertial sensors are a popular choice for human motion tracking due to their small size, light weight, and high accuracy (100). A recent study developed several supervised learning classifiers, using linear discriminant analysis, support vector machines, decision tree, random forests, and multilayer perceptrons, to discriminate spasticity levels of elbow muscle group based on data from three inertial sensors placed on the dorsal side of the elbow (94). The results showed that machine learning algorithms based on inertial data performed well in classification spasticity of the elbow muscle with the accuracy of 95.4%. Additionally, visual biofeedback was added to the inertial sensor-based device to provide additional information such as abnormal muscle response in addition to the passive stretching velocity of the lower extremities (95). The device has good test-retest reliability in knee flexor and extensor, and ankle plantar-flexor (ICCs > 0.8).

Robot-aided spastic devices may improve the accuracy of spasticity assessment and also can be used in clinical settings for patient-specific rehabilitation (91). Robot-assisted devices are often developed based on a variety of equipment such as goniometers, pressure sensors and EMG electrodes (101). A recent study found that it is possible to automate the assessment of spasticity using robotic exoskeletons (96). The robot can assess upper limb spasticity under several active- or passive-motion conditions, such as recording elbow joint angles and flexion and extension torques. For example, a pediatric exoskeleton was used to assess hip and knee flexor and extensor spasm while standing in children with CP (97). The exoskeleton measured subject's joint resistance to passive movements at controlled velocities. The inter-rater reliability is better during fast and medium movement speed compared to slow speeds in assessing lower limb spasticity with the ICC ranged between 0.32 to 0.70 (all *p* ≤ 0.01). It has been suggested that automatically robot-assisted devices could be alternatives for clinical spasticity evaluation.

Myotonometry is a new technology in quantifying spasticity by investigating the pathophysiological mechanisms of spastic muscles (10, 92). Myotonometry differs from traditional stretch techniques, using a myotonometry probe to send many brief pulses to spastic muscles, rather than to detect the change in resistance during passive stretching (4). Rydahl and Brouwer found that the ankle stiffness, measurement by myotonometry, in patients with chronic stroke is significantly higher (*P* < 0.02) than in healthy individuals (102). Yamaguchi et al. evaluated passive muscle-tendon-joint stiffness, reflex mediated stiffness and range of movement using myotonometry for spastic patients and demonstrated a good to excellent inter- and intra-rater reliability (ICCs = 0.62~0.91) (98). However, Li et al. compared the validity of myotonometry and passive stretch measurements in spasticity assessment and found significant negative relationships between the stretch test and the myotonometer measurements (*r* < −0.5, *p* < 0.05) (92). More studies are needed to evaluate the effectiveness of myotonometry.

Although these instruments (wearable sensor, robot-assisted devices and myotonometry) based on spasticity assessment have shown reliability. However, they are not as widely used as clinical scales. The main limitation is the lack of well-equipped laboratories and experienced practitioners. In addition, these devices may not be as suitable for clinical settings. For example, this may increase the burden on clinical physiotherapists learning to operate spasticity assessment equipment and may be more suitable for scientific studies to explore the pathological mechanism of spasticity rather than for outpatient spasticity assessment.

## Repetitive peripheral magnetic stimulation

Electrical stimulation is often used to stimulate muscles and nerves. Motion can be stimulated by providing enhanced sensory input to the paretic limb (103). High frequency repetitive peripheral magnetic stimulation (rPMS) can induce muscular contractions by stimulating of the terminal branches of motor nerves (104) (see Table 5). rPMS has been successfully applied in neurologically impaired adults and CP children to assess spasticity, to improve ranges of motions and motor functions (112, 113). Han et al. compared the effect between magnetic and electrical stimulation and found that the average maximum peak torque from each subject induced by magnetic stimulation is higher than that of electrical stimulation (9.5 ± 4.8 vs. 4.4 ± 2.9 Nm) (114).

**Table 5:** Other spasticity assessment methods.

**Methods**	**Compared tests**	**Subjects (sample size)**	**Muscle groups**	**Results**	**Characteristics**	**References**
rPMS	MAS; MTS FMS; PMS	Stroke (32) Stroke/normal (24/12)	EF; EE; SF; SE; WF	Reduce spasticity; very good test-retest reliability (ICC = 0.85~0.99); reduced amplitude, velocity and acceleration of rPMS-induced movement for stroke group	Painless and non-invasive; can assess and treat spasticity; induce muscle contractions	(104, 105)
NMSM	GMFCS; MTS	CP/TD (11/9)	HAM; VAS; RF; BFS; GAS	Predict knee angle, muscle activity, and fiber length and velocity; hamstrings of CP are stiffer than TD	Predict muscle behavior at different speeds	(106)
	MAS; EMG	Stroke/normal (3/3)	EF; EE	EF activated small; only one EMG parameter need to be adjusted in this model compared other model	Uses muscle activation and musculotendon dynamics to calculate internal torque	(107)
	MAS	Stroke (18)	EF; EE	Clinicians can perceive the resistance from the simulator, but larger than actual resistance	Building spasticity simulator; mimics and measure resistance and joint motion according to patients' actual response	(108)
Tele-medicine	MAS	Spasticity/normal (26/35)	WF; WE; SA; AF	94% agreement between two tele-neurologists in remote screening spasticity (kappa = 0.875)	Telemedicine and in-person screen spasticity	(109)
	PROM; MAS	Stroke (12)	EF	The agreements for the strength and the spasticity of EF between in-person and remote assessments are (*k =* 0.643) and (*k =* 0.308), respectively	In-person vs. remotely assessment for spasticity	(110)
	QOL	Stroke/TBI (123/28)	EF	72.2% of patient-perceived increase in spasticity and 72.9% perceived decrease of quality of life; 7.3% of patients use telemedicine tools	Self-report based on questionnaire	(111)

rPMS, repetitive peripheral magnetic stimulation; NMSM, neuromusculoskeletal model; FMS, Fugl–Meyer scale; PMS, passive motion stretch; MAS, modified Ashworth Scale; MTS, modified Tardieu Scale; GMFCS, Gross Motor Function Classification System; EMG, electromyography; PROM, passive range of motion; QOL, quality of life; CP, cerebral palsy; TD, typically developing children; TBI, Traumatic brain injury; EF, elbow flexor; EE, elbow extensor; SF, shoulder flexor; SE, shoulder extensor; HAM, hamstring; VAS, vastus lateralis, medialis and intermedius; RF, rectus femoris; BFS, biceps femoris short head; GAS, gastrocnemius; WE, wrist extensor; SA, shoulder adductor; AF, ankle dorsiflexor.

rPMS is an effective approach since some patients with severe spasticity are unable to perform sustained voluntary-contraction movements (105). rPMS can activate specific muscle groups such as quadriceps (114) and elbow flexors (115) by using a figure-of-eight coil. Researcher assessed spasticity of wrist flexors by calculating the difference between the maximum passive ROM and the rPMS-induced movement and showed a good test-retest reliability (ICC > 0.85) (105).

Numerous studies have also explored the effect of rPMS on rehabilitation, in which rPMS can significantly reduce spasticity in patients with central nervous injuries regardless of single or multiple stimulations (116, 117). Conforto et al. found that rPMS can improve motor performance of the paretic upper limb in patients with stroke, and has no reported serious adverse events such as pain and joint deformity (103). The evaluation protocol of rPMS is similar to MTS, involving ROM measurement. The main difference between rPMS and MTS is that rPMS can induce physiological muscle contraction rather than passive stretches (105). Use of rPMS will predictably improve spasticity assessment and rehabilitation in the future.

It is should be noted that current studies using rPMS in the assessment of spasticity are limited to the upper extremities, and rPMS may not be appropriate for the assessment of patients with lower limb spasticity. Although current studies have demonstrated its reliability in the assessment of upper extremity spasticity, validation in the assessment of lower extremity spasticity is also needed.

## Neuro-musculoskeletal model-based spasticity assessment

Neuromusculoskeletal models could gain insights into the underlying pathology of spastic muscles (118) (see Table 5). Analyzing spastic muscle behaviors using neuromusculoskeletal model could yield valuable information on tissue and muscle reflex activities (106). However, developing valid neuromusculoskeletal models is very challenging due to the complexity of spasticity muscle behaviors. Moreover, evaluating the neuromusculoskeletal models are also complex, need validating and training with several types of experimental data (e.g., joint angle, angular velocity, resistance or passive ROM) (119).

van der Krogt et al. conducted spasticity and contracture assessment of the hamstring muscles in children with CP using a neuromusculoskeletal model (106). The model is modified from Gait 2392 in OpenSim platform. The model is used to evaluate the left knee, which could move freely, and all muscles were removed except for those around the left knee. Researchers simulated muscle spasticity behavior during slow and fast passive stretches using forward dynamics algorithms based on the modified model. During on the simulation, sEMG signals recorded are used as the input and the internal torques are calculated using muscle activation dynamics and muscle-tendon dynamics (106, 107). A new study has introduced a neuromusculoskeletal model to simulate a passive wrist extension test to evaluate the neural and non-neural properties of spastic wrist flexors by modeling the stretch reflex pathway (120). The results showed that patients with moderate and severe spasticity had significantly higher stiffness than controls.

A recent study simulated actual spasticity responses in patients with stroke based on a haptic model of MAS (108). This requires the use of MAS to assess patient's spasticity response involving resistance and joint motion. Each grade of MAS was quantified by using three spasticity parameters (e.g., catch angle index, catch magnitude index and post-catch torque shape index) (108). A model of MAS elbow spasticity was developed based on the parameters. The results showed that the duration of the catch was successfully mimicked by two experienced clinicians, but not the magnitude of elbow resistance. The elbow model may lack reliability in spasticity measurement and thus further investigation is needed. Therefore, the existing results show that the musculoskeletal model is feasible to evaluate spasm, but at present, most of the research is focused on scientific research, and few clinical reports.

## Telemedicine based spasticity assessment

The COVID-19 pandemic has impacted the field of physical medicine, especially in the spasticity outpatient evaluation, making the diagnosis more difficult. The emergence of telemedicine may be one of the solutions to solve this dilemma (see Table 5). A recent study introduced the procedure of conducting an outpatient telemedicine rehabilitation or rehabilitation visit based on a virtual framework in which the clinician guides the caregiver through using tele-communication technologies in the evaluation of spasticity (121).

A number of studies have focused on and investigated the reliability of tele-evaluation of spasticity. Harper et al. (109) used telemedicine technology to screen spasticity and compared the results with in-person evaluation. They found that telemedicine assessment is similar to in-person evaluation (e.g., two examiners in this study) with the accuracy of 94% (kappa = 0.875, 95% CI: 0.640–1.000). Verduzco-Gutierrez et al. guided caregivers through remote access technology for outpatient assessment of spasticity (122). The evaluation includes active or passive motions as well as functional movement tasks, quality of life and self-report spasticity assessment (122). However, the tele-assessment of spasticity lacks reliability verifications and only some non-urgent evaluations were performed such as routine follow-up.

Kim et al. discovered that the agreements for the strength and the spasticity of elbow flexor between in-person and remote assessments were substantial (kappa = 0.643) and fair (kappa = 0.308), respectively (110). De Donno et al. showed that tele-evaluation of spasticity is inadequate and only 7.3% of patients can be accessed, as it involves a lot of ethical, medico-legal and technical issues (111).

A recent study summarized the potential, challenges and recommendations for telemedicine in long-term neurological diseases and investigated how telemedicine can be used effectively (123). Patients can easily access medical services through tele-communication technology during the COVID-19 pandemic, and look forward to continue to develop after the pandemic ends. The development of telemedicine needs multilateral cooperation among patients, caregivers and health care professionals especially with government support. Therefore, expanding the use of telemedicine has profound implication for those with spasticity who have mobility impairments. The current use of telemedicine to assess spasticity lacks systematic procedures and checklists. More studies on the validity and reliability of telemedicine in spasticity assessment are needed in the future. It is important to note that a telemedicine assessment requires the participation of several people, such as a guardian or caregiver who are remotely guided by a physical therapist. It may be more suitable for bedridden patients with spasticity. In addition, there is no effective consensus on telemedicine assessment as a transitional method for the assessment of spasticity in the context of the COVID-19 pandemic. The telemedicine assessment mechanism should be further improved in the future.

## Conclusion

This review article has presented an overview the methods developed for the assessment of spasticity from 2016 to 2022 and explores their feasibility. The newly developed spasticity methods aim to standardize experimental protocols and outcome measures, enabling quantified, accurate, and intelligent assessment. However, it is important to note that spasticity patients often exhibit more pronounced pathological during active movements rather than passive movements. The current evaluation methods tend to prioritize passive evaluation while overlooking assessment during active motion. Therefore, future research should focus on combining active and passive motor assessments and incorporating patient self-reports to provide a comprehensive evaluation of spasticity. By considering both active and passive aspects, we can obtain a more holistic understanding of spasticity and improve the accuracy of assessment. Further exploration in this direction will contribute to advancing the field of spasticity assessment.

## Author contributions

Conceptualization: YM and MH. Methodology: YM and DL. Formal analysis: JH, AL, JY, and CQ. Writing: JH and YM. Review and editing and funding acquisition: YM, MH, and DL. All authors have read and agreed to the published version of the manuscript. All authors contributed to the article and approved the submitted version.
